# Body mass index associated with monoclonal gammopathy of undetermined significance (MGUS) progression in Olmsted County, Minnesota

**DOI:** 10.1038/s41408-022-00659-9

**Published:** 2022-04-19

**Authors:** Geffen Kleinstern, Dirk R. Larson, Cristine Allmer, Aaron D. Norman, Grace Muntifering, Jason Sinnwell, Alissa Visram, Vincent Rajkumar, Angela Dispenzieri, Robert A. Kyle, Susan L. Slager, Shaji Kumar, Celine M. Vachon

**Affiliations:** 1 School of Public Health, University of Haifa, Haifa, Israel; 2 Department of Quantitative Health Sciences, Mayo Clinic, Rochester, MN USA; 3 University of Wisconsin, La Crosse, WI USA; 4 Department of Medicine, Division of Hematology, Mayo Clinic, Rochester, MN USA; 5 The Ottawa Hospital, Ottawa Hospital Research Institute, Ottawa, ON Canada

**Keywords:** Risk factors, Cancer

## Abstract

Monoclonal gammopathy of undetermined significance (MGUS) is a premalignant clonal disorder that progresses to multiple myeloma (MM), or other plasma-cell or lymphoid disorders at a rate of 1%/year. We evaluate the contribution of body mass index (BMI) to MGUS progression beyond established clinical factors in a population-based study. We identified 594 MGUS through a population-based screening study in Olmsted County, Minnesota, between 1995 and 2003. Follow-up time was calculated from the date of MGUS to last follow-up, death, or progression to MM/another plasma-cell/lymphoid disorder. BMI (kg/m^2^ < 25/≥25) was measured close to screening date. We used Cox regression to estimate hazard ratios (HR) and 95% confidence intervals (CI) for the association of BMI ≥ 25 versus BMI < 25 with MGUS progression and also evaluated the corresponding c-statistic and 95% CI to describe discrimination of the model for MGUS progression. Median follow-up was 10.5 years (range:0–25), while 465 patients died and 57 progressed and developed MM (*N* = 39), AL amyloidosis (*N* = 8), lymphoma (*N* = 5), or Waldenstrom-macroglobulinemia (*N* = 5). In univariate analyses, BMI ≥ 25 (HR = 2.14,CI:1.05–4.36, *P* = 0.04), non-IgG (HR = 2.84, CI:1.68–4.80, *P* = 0.0001), high monoclonal (M) protein (HR = 2.57, CI:1.50–4.42, *P* = 0.001), and abnormal free light chain ratio (FLC_r_) (HR = 3.39, CI:1.98–5.82, *P* < 0.0001) were associated with increased risk of MGUS progression, and were independently associated in a multivariable model (c-statistic = 0.75, CI:0.68–0.82). The BMI association was stronger among females (HR = 3.55, CI:1.06–11.9, *P* = 0.04) vs. males (HR = 1.39, CI:0.57–3.36, *P* = 0.47), although the interaction between BMI and sex was not significant (*P* = 0.15). In conclusion, high BMI is a prognostic factor for MGUS progression, independent of isotype, M protein, and FLC_r_. This association may be stronger among females.

## Introduction

Monoclonal gammopathy of undetermined significance (MGUS) is a premalignant clonal disorder [1, 2] characterized by the presence of an M-protein in serum without evidence of multiple myeloma (MM) or other lymphoproliferative diseases [3]. MGUS progresses at a rate of 1% per year to MM, or other plasma-cell or lymphoid disorders [2, 4], and occurs in 3.2% and 5.3% of persons 50 and 70 years of age or older, respectively [5]. Clinical factors consistently reported to be associated with MGUS progression include MGUS isotype, abnormal serum free light-chain ratio (FLCr), and high serum monoclonal protein (M protein) level (≥1.5 g per deciliter) [5, 6]. Although body mass index (BMI) or obesity has been found to be associated with MM risk [7–16], and was concluded to be a “preventative factor” for MM by the International Agency for Research on Cancer (IARC) [17], studies of BMI measures with MGUS are limited and inconsistent [13, 18–20]. Only two studies examined obesity and MGUS transformation to MM and other lymphoid plasma cell disorders and found positive associations with overweight and obesity; [13, 19] one study found no evidence of an association between obesity and MGUS progression [21]. However, BMI has not been evaluated in the context of established clinical prognostic factors [8, 19, 22–26] for MGUS progression. Here, we evaluate the contribution of BMI to MGUS progression beyond clinical prognostic factors in a population-based study and examine differential associations by sex.

## Methods

### Study population

We studied 594 patients residing in Olmsted County Minnesota, who were identified with MGUS in a screening study conducted between 1995 and 2003 at the Mayo Clinic [5]. Patients with light-chain MGUS were not included. This study was approved by the Mayo Clinic Institutional Review Board.

### Prognostic factors

The following factors were evaluated with risk of MGUS progression: age, sex, M-protein level (<1.5 g/dl or ≥1.5 g/dl), FLC_r_ [normal (0.26–1.65) or abnormal (<0.26 and/or >1.65)], isotype (IgG, IgM, IgA, biclonal), and BMI (kg/m^2^) which was calculated using height and weight values in the clinical record close to the screening date (80% of patients had measures within 2 years), and categorized into BMI < 25 and BMI ≥ 25. We also evaluated BMI as a continuous variable per 5 BMI units. BMI within a year of last follow-up or diagnosis date was also available on a subset. Suppressed uninvolved immunoglobulins (0, vs 1+) were also assessed with MGUS progression in secondary analyses, as this variable was only available on a subset (Table 1). Patients with biclonal gammopathy were excluded from all the analyses that were performed according to isotype, and participants were categorized by IgG and non-IgG (IgM or IgA).

**Table 1:** Clinical characteristics of MGUS patients in the entire cohort and by sex.

	Entire cohort (*N* = 594)		Male (*N* = 301)		Female (*N* = 293)	
Risk factor	*N*	%	*N*	%	*N*	%
*Follow-up*						
person-yrs	6846		3522		3325	
Median (yrs)	10.5		10.5		10.6	
Range (yrs)	0–24.9		0–24.9		0–24.5	
*Age at screening*						
Median (yrs)	73		71		75	
Range (yrs)	50–98		52–95		50–98	
<65	157	26.4	96	31.9	61	20.8
≥65	437	73.6	205	68.1	232	79.2
*Isotype*						
IgG	412	69.3	206	68.4	206	70.4
IgM	102	17.2	50	16.6	52	17.7
IgA	66	11.1	34	11.3	32	10.9
Biclonal	14	2.4	11	3.7	3	1.0
*M protein*						
<1.5 g/dl	432	77.7	222	77.9	210	77.5
≥1.5 g/dl	124	22.3	63	22.1	61	22.5
Missing	38		16		22	
*Free light-chain ratio*						
Normal (0.26–1.65)	375	72.1	190	72.5	185	71.7
Abnormal (<0.26 or >1.65)	145	27.9	72	27.5	73	28.3
Missing	74		39		35	
*Uninvolved immunoglobulins*						
0	350	83.5	176	84.2	174	82.9
≥1	69	16.5	33	15.8	36	17.1
Missing	175		92		83	
*Body mass index (kg/m*^*2*^*)*						
Median	26.8		27.3		26.2	
Range	13.9–58.9		16.2–43.0		13.9–58.9	
Missing	39		19		20	
<25	181	32.6	73	25.9	108	39.6
≥25	374	67.4	209	74.1	165	60.4
*Race*						
White	493	83.0	253	84.1	240	81.9
Black	2	0.3	2	0.6	0	
Asian	3	0.5	3	1	0	
Unknown/missing	96	16.2	43	14.3	53	18.1

### Statistical analysis

Follow-up time was calculated from the date of MGUS screening to date of last follow-up, death, or progression to MM or another plasma-cell or lymphoid disorder. We used Cox regression to estimate the association of BMI with risk of MGUS progression to MM or another plasma-cell or lymphoid disorder, univariately, and accounting for clinical factors using a multivariable model. Analyses were also stratified by sex, and interaction between BMI and sex were evaluated by inclusion of interaction term of BMI (as a binary variable) and sex into the multivariable model. We also evaluated the interaction between the other known prognostic factors: isotype (as a binary variable IgG/non-IgG), high M protein level, and abnormal FLC_r_, and sex, by inclusion of interaction term of each prognostic factor and sex into the multivariable model. In a sensitivity analysis, we ran the univariate models restricted to patients with complete data on all clinical factors, as included in the multivariable model. We also conducted two sets of exploratory analyses given the smaller sample size. We examined the impact of suppressed uninvolved immunoglobulins and the change in BMI from baseline to follow-up or diagnosis date. We report hazard ratios (HR) and 95% confidence intervals (CI). To evaluate model discriminative ability, we computed a *c*-statistic and 95% CI [27] for the adjusted Cox regression models. The *c-*statistic is equivalent to the area under the ROC curve and is the probability that the measure or predicted risk is higher for a case who experiences the outcome of interest (in our case MGUS progression to MM or another plasma-cell or lymphoid disorder), and a case who does not [28]. A *c*-statistic = 0.5 is equivalent to chance; *c*-statistic > 0.7 is a good discrimination between cases who experience the outcome and cases who do not; *c*-statistic > 0.8 signifies a strong discrimination; and *c*-statistic = 1 indicates perfect discrimination [29].

The risk of progression to each of the following diseases: MM, lymphoma, chronic lymphocytic leukemia, and Waldenström’s macroglobulinemia, as compared with the risk in the general population was determined by applying age- and sex-specific incidence rates for these conditions in the cohort of white participants from the Iowa Surveillance, Epidemiology, and End Results (SEER) program [30] to the age-, sex-, and calendar year-specific number of person-years of follow-up in our study cohort, as previously described [2]. The age- and sex-specific incidence rates for AL amyloidosis were based on data from Olmsted County, Minnesota, since these rates were not included in the SEER program [31]. Relative risks (RR) were calculated and the respective confidence intervals were based on a Poisson approach [32]. Kaplan Meier curves are presented by BMI (<25, ≥25) for the overall cohort and stratified by sex, after accounting for death as a competing risk. The cumulative incidence of progression was calculated both with and without accounting for death as a competing risk. Statistical code is available upon request.

### Data sharing

Deidentified data can be shared with appropriate data use agreement and IRB approvals.

## Results

### Characteristics of the MGUS patients at the screening date

Of the 594 MGUS patients, 83% were white, 51% were male, and the median age at the screening date was 73 (range:50–98 years) with 74% being 65 years or older. The immunoglobulin type was IgG in 69.3% of patients, IgM in 17.2%, IgA in 11.1%, and biclonal in 2.4%. High M protein level was observed in 22% and abnormal FLC_r_ in 28%. The median BMI was 26.8 (range:13.9–58.9), where BMI ≥ 25 was observed in 67% of patients (Table 1).

### MGUS progression to MM, or other plasma-cell or lymphoid disorders

The median follow-up time was 10.5 years (range:0–25). Over a median of 6846 person-years, 465 patients (78%) died and 57 patients progressed and developed MM (*N* = 39), amyloidosis (*N* = 8), Waldenstrom’s macroglobulinemia (*N* = 5) or other lymphoma (*N* = 5). Sixty percent (*N* = 34) of those who progressed were male. The risk of developing these disorders was 49.9 times (95% CI:37.8–64.7) higher risk compared to age- and sex-matched background population (Table 2).

**Table 2:** Risk and type of progression in the overall MGUS cohort.

Outcome	Observed	Expected	RR	95% CI
Any progression	57	1.14	49.9	(37.8, 64.7)
Multiple myeloma	39	0.15	257.9	(183.4, 352.6)
Lymphoma	5	0.67	7.5	(2.4, 17.4)
Waldenström’s macroglobulinemia	5	0.02	292.2	(94.9, 681.9)
Chronic lymphocytic leukemia	0	0.24	0.0	(0.0, 15.3)
AL amyloidosis	8	0.06	128.3	(55.4, 252.9)

The cumulative risk of progression to one of these disorders (not accounting for death due to competing risk) was 3% at 5 years, 10% at 10 years, 14% at 15 years, and 17% at 20 years. The overall rate of progression was 0.87 events (CI:0.67–1.12) per 100 person-years (Supplementary Table 1). The rate of progression was 1.01 (CI:0.73–1.42) events per 100 person-years in males, as compared with 0.72 (CI:0.48–1.08) events per 100 person-years in females, however, the difference was not statistically significant (*P* = 0.24). The risk factors for progression in the overall cohort and by sex are shown in Supplementary Table 1.

### Risk of MGUS progression by BMI and sex

**Fig. 1:**
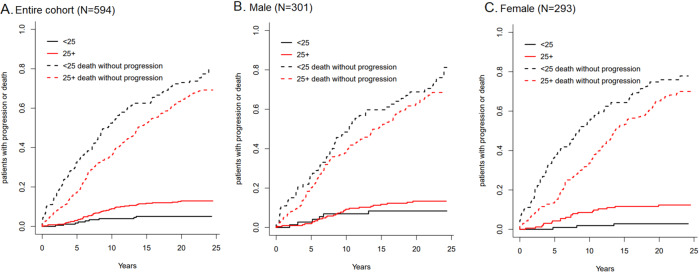
Cumulative Incidence of progression of MGUS by BMI, with death accounted for as a competing risk. **A**. Entire cohort (*N* = 594); **B**. Males (*N* = 301); **C**. Females (*N* = 293). BMI stratified by <25 (black solid line), <25 death without progression (black dashed line), 25+ (red solid line), and 25+ death without progression (red dashed line).

The cumulative risk of progression to MM, or other plasma-cell or lymphoid disorders (not accounting for death due to competing risk) for MGUS patients with BMI < 25 was 2.7% at 5 years, 6.3% at 10 years, 9.3% at 15 years, and 11.8% at 20 years; while for patients with BMI ≥ 25 it was higher: 3.7% at 5 years, 12.6% at 10 years, 17.7% at 15 years, and 22.0% at 20 years (Table 3); the difference was statistically significant (log rank *P* = 0.03). A similar trend is observed when stratifying by sex, although the difference was statistically significant for females (*P* = 0.03) and not for males (*P* = 0.46) (Table 3). Figure 1 shows the cumulative risk of progression when accounting for death as a competing risk among patients with BMI < 25 and BMI ≥ 25, as well as stratified by sex; the cumulative risk of progression when accounting for death as a competing risk was lower both for BMI < 25 and BMI ≥ 25, however, the difference was still statistically significant (*P* = 0.006). A similar trend is observed when stratifying by sex, although the difference was statistically significant for females (*P* = 0.007) and not for males (*P* = 0.31) (Table 3).

**Table 3:** Cumulative incidence rates of MGUS progression by BMI and sex.

	Accounting for death as a competing risk				Without accounting for death			
BMI\Years	5	10	15	20	5	10	15	20
*Entire cohort*								
<25	1.7%	3.9%	5.1%	5.1%	2.7%	6.3%	9.3%	11.8%
≥25	3.0%	9.0%	11.7%	12.9%	3.7%	12.6%	17.7%	22.0%
*Male*								
<25	2.7%	6.9%	8.4%	8.4%	3.3%	9.3%	13.3%	13.3%
≥25	1.9%	9.3%	11.8%	13.4%	2.1%	13.0%	18.0%	22.4%
*Female*								
<25	0.94%	1.9%	2.8%	2.8%	1.4%	3.3%	5.9%	5.9%
≥25	4.3%	8.6%	11.6%	12.3%	4.8%	10.5%	16.0%	18.6%

### Modeling independent prognostic factors for MGUS progression

In univariate analyses, BMI ≥ 25 (HR = 2.14, CI:1.05–4.36, *P* = 0.04), non-IgG isotype (HR = 2.84, CI:1.68–4.80, *P* = 0.0001), high M protein (HR = 2.57, CI: 1.50–4.42, *P* = 0.001), and abnormal FLC_r_ (HR = 3.39, CI:1.98–5.82, *P* < 0.0001) were associated with increased risk of progression (Table 4). These four factors were independently associated with MGUS progression in a multivariable model, with a c-statistic = 0.75 (CI:0.68–0.82). When stratifying by sex, associations of BMI with progression were stronger among females (adjusted HR = 3.46, CI:1.02–11.8, *P* = 0.047) than males (adjusted HR = 1.13, CI:0.42–2.99, *P* = 0.88); corresponding models had c-statistics of 0.81 (CI:0.72–0.90) vs 0.71 (CI:0.60–0.82) respectively (Table 4). However, a test for interaction between BMI and sex was not statistically significant (*P* = 0.15). When using a continuous BMI in the multivariable model (Supplementary Table 2), we also found a significant association per 5 BMI units among females (HR = 1.49, CI:1.02–2.17), but not among males (HR = 1.12, CI:0.75–1.68).

**Table 4:** Factors associated with risk of MGUS progression.

			Univariate			Univariate^a^					Multivariable^b^		
	*N*	Event	HR	95% CI	*P*	*N*	Event	HR	95% CI	*P*	HR^a^	95% CI	*P*
*BMI (≥25)*													
Overall	555	56	2.14	1.05–4.36	0.04	446	49	2.07	0.97–4.42	0.06	1.92	0.90–4.11	0.09
Male	282	33	1.39	0.57–3.36	0.47	223	27	1.21	0.46–3.21	0.70	1.13	0.42–2.99	0.81
Female	273	23	3.55	1.06–11.9	0.041	223	22	3.49	1.03–11.8	0.044	3.46	1.02–11.8	0.047
*Isotype (non-IgG)*													
Overall	580	56	2.84	1.68–4.80	0.0001	446	49	3.22	1.83–5.65	<0.0001	3.17	1.79–5.60	<0.0001
Male	290	33	2.50	1.25–4.99	0.01	223	27	2.64	1.22–5.70	0.01	2.99	1.17–5.71	0.02
Female	290	23	3.44	1.52–7.82	0.003	223	22	4.11	1.77–9.58	0.001	3.79	1.58–9.07	0.003
*M protein (≥1.5* *g/dl)*													
Overall	556	55	2.57	1.50–4.42	0.001	446	49	2.50	1.41–4.45	0.002	2.22	1.23–3.99	0.008
Male	285	33	2.03	0.99–4.20	0.055	223	27	1.70	0.75–3.90	0.21	1.64	0.70–3.84	0.26
Female	271	22	3.64	1.57–8.41	0.003	223	22	3.84	1.66–8.86	0.002	3.20	1.36–7.55	0.008
*FLC*_*r*_ *(Abnormal)*													
Overall	520	53	3.39	1.98–5.82	<0.0001	446	49	3.49	1.99–6.12	<0.0001	2.58	1.45–4.59	0.001
Male	262	30	2.32	1.13–4.77	0.023	223	27	2.47	1.16–5.29	0.02	1.96	0.89–4.32	0.10
Female	258	23	5.72	2.42–13.5	<0.0001	223	22	5.47	2.29–13.1	0.0001	3.97	1.64–9.59	0.002
*C-statistic*													
Overall											0.75	0.68–0.82	
Male											0.71	0.60–0.82	
Female											0.81	0.72–0.90	

^a^Also adjusted for age at MGUS screening date.

^b^For overall: model includes 446 MGUS cases (with 49 events) who had complete clinical data available; for male: model includes 223 MGUS cases (with 27 events) who had complete clinical data available; for female: model includes 223 MGUS cases (with 22 events) who had complete clinical data available.

Univariate and multivariable Cox regression models.

Interestingly, in the multivariable model, the other known prognostic factors: non-IgG isotype (HR_female_ = 3.79, HR_male_ = 2.99, P_interaction_ = 0.46), high M protein (HR_female_ = 3.20, HR_male_ = 1.64, *P*_interaction_ = 0.16), and abnormal FLC_r_ (HR_female_ = 3.97, HR_male_ = 1.96, *P*_interaction_ = 0.16) also showed a stronger association in females compared to males, although the interaction between sex and each of these prognostic factors were not statistically significant (all *P* > 0.05).

In an exploratory analysis we also found suppressed uninvolved immunoglobulins (1 + versus 0) associated with increased risk of progression in a univariate (HR = 4.90, CI:2.71–8.86; *n* = 410), and multivariable model adjusting for isotype, M-protein, FLC_r_ and BMI (HR = 3.77, CI:1.92–7.41; *n* = 358) (Supplementary Table 3). Although sample size was limited for sex-specific associations, there was suggestion of a stronger association of uninvolved immunoglobulins with progression among males (adjusted HR = 5.00, CI:2.06–12.1, *P* < 0.0001) than females (adjusted HR = 1.83, CI:0.63–5.34, *P* = 0.27); but the interaction with sex was not significant (*P* = 0.26).

BMI within one year of follow-up or diagnosis date was available on 438 of the MGUS; the proportion of those who had low BMI (<25) at both time points was 28%, while 44% had high BMI (≥25) at both time points. Only 5% decreased in BMI over time and 23% increased over time (Supplementary Table 4). Adjusted for age and sex, high BMI ≥ 25 at both baseline and follow-up had the highest risk of progression (HR = 2.73; CI:0.1.20–6.24) compared to MGUS who had lower BMI at both time points (Supplementary Table 4). Associations for BMI < 25 at baseline but higher at follow-up (HR = 1.17; CI: 0.24–5.70) and those who started with high BMI but decreased at follow-up (HR = 0.66; CI:0.21–2.07) were not significant. Further adjustments for prognostic factors in a small sample (*N* = 362) did not change the findings for those with high BMI at both time points (Supplementary Table 4). Power limited examination of differences by sex.

## Discussion

In this study, we found evidence that high BMI is associated with MGUS progression, independent of isotype, M protein level, FLCr, and uninvolved immunoglobulins. We also found suggestive evidence that this was driven by the effect in females rather than males, although we were limited in power.

Few studies have examined whether obesity affects the transformation of MGUS into MM [13, 19]. A retrospective study of 7878 MGUS patients with a median follow up of 5.7 years reported an increased risk of transformation from MGUS to MM among overweight (BMI: 25–29.9) and obese (BMI ≥ 30) MGUS patients compared to normal weight patients, after adjusting for race (white, black, other), marital status, income quartile, Charlson comorbidity index excluding diabetes, presence of diabetes, and serum creatinine concentration at baseline [13]. In a population-based study from Iceland of 5725 individuals, 300 were identified with MGUS and 29 with MM or other LP diseases; they evaluated 11 different obesity measures and found that high BMI (≥25) measured at midlife was associated with an increased risk of progression from MGUS to MM and other LP diseases after adjusting for age and sex [19]. However, the results of this study were limited due to the small number of MM and other LP diseases [19]. In contrast, a case-control study conducted at the Mayo Clinic, Rochester, Minnesota, of 100 MGUS patients who progressed to MM or related malignancy, and 100 controls with MGUS who did not progress, found that obesity (defined as BMI > 30) did not have a significant effect on MGUS progression (OR = 0.66, 95% CI:0.36–1.22) [21]. Moreover, a cross-sectional study with 40 MGUS and 32 newly diagnosed MM patients found that those with MM had higher abdominal fat cross-sectional areas and higher fat metabolic activity compared to patients with MGUS. They suggested that these parameters may serve as novel biomarkers of progression of MGUS to MM [33].

Past studies have shown that BMI or obesity contributes to an increased risk for MM [7–17] and MGUS, although the latter is inconsistent [13, 18–20]. It is difficult to conclude from these studies whether BMI is associated with the initiation of MGUS or the increased risk of transformation from MGUS to MM or other LP diseases. However, our current findings coupled with the two other studies described above [13, 19] provide evidence that high BMI is associated with MGUS progression to MM or other plasma-cell or lymphoid disorders. We further note that this association is independent of other prognostic factors.

Moreover, this study is the first known to investigate sex differences in evaluating prognostic factors among MGUS patients. We found that the established prognostic factors such as isotype, M protein level, and FLCr had a stronger association, indicated by larger effect size, in females compared to males, which was also seen for high BMI; exploratory analyses suggested uninvolved immunoglobulins had a stronger association in males compared to females but sample size was limited. However, the interactions between sex and each of those factors were not statistically significant, likely due to insufficient power. Analyses of change in BMI from baseline to follow-up found those who were high at both time points had the greatest risk of progression relative to those who remained in the lower BMI category at both time points. There was a non-significant decreased risk among those who had decreased BMI over time, but these analyses and their interpretation were limited by power. Future studies should investigate potential sex differences as well as examine changes in BMI over time with MGUS progression, also in context of changes in prognostic factors, to support weight loss recommendations, in particular for women with MGUS.

Strengths of this study include the study design of a population-based screening study, with consistently ascertained isotype, M protein, FLCr; also, weight and height were directly measured in the clinical practice. This is the first study to evaluate the contribution of BMI to MGUS progression beyond the known clinical prognostic factors and found a high discrimination (c-statistic = 0.75). Limitations of this study include the lack of power for analyses of sex differences across the various prognostic factors, including BMI and BMI change. Twenty percent of MGUS patients did not have BMI measurement within 2 years from MGUS screening. Moreover, BMI does not provide information on fat distribution and cannot evaluate lean and fat mass; however, prior studies have reported that BMI is correlated with body composition and fat distribution [30]. Our results of higher risk of progression for those with high BMI across time should be viewed as preliminary but support a role of chronic inflammation, through obesity, as a risk factor for MGUS progression. We had a large number of deaths prior to opportunity for progression to MM and other plasma-cell or lymphoid disorders among our MGUS patients, and our data and analyses reflect the competing risk of death due to other causes. The majority of MGUS patients in this study (83%) were white and therefore our results may not be applicable to other races, such as those with African ancestry who are known to be at higher risk for MGUS and MM compared to other races [34, 35].

Future studies should investigate immune markers and their changes, associated with obesity, such as reduced expression of adiponectin which has previously found to be associated with progression from MGUS to MM [36, 37]. This may provide information on mechanisms through which BMI may influence MGUS progression, in particular among women. In addition, well-powered studies are needed on sex differences in prognostic factors among MGUS patients, and particularly BMI, as well as to assess whether changes in BMI influence MGUS progression. These findings may improve MGUS risk stratification and define a targeted group of MGUS patients with obesity or high BMI for weight loss intervention, to reduce the risk of transformation to MM or other plasma-cell or lymphoid disorders.

## Supplementary information

Supplemental Tables

Check List
