# Supplementary material for: Body mass index associated with monoclonal gammopathy of undetermined significance (MGUS) progression in Olmsted County, Minnesota
Source: Blood Cancer J. 2022 Apr 19;12(4):67. doi: 10.1038/s41408-022-00659-9 (PMC9018764; doi:10.1038/s41408-022-00659-9)
Supplement: Supplementary file 1 — Supplemental Tables [file 41408_2022_659_MOESM1_ESM.docx]

**Supplementary Table 1.** Rate of Progression of Monoclonal Gammopathy of Undetermined Significance (MGUS) per 100 Person-Years in the entire cohort and by sex

|  |  | Entire cohort (N=594) | | | | Male (N=301) | | | | Female (N=293) | | | |
| --- | --- | --- | --- | --- | --- | --- | --- | --- | --- | --- | --- | --- | --- |
| Risk factor | Risk factor | N | Events | Number of  progression per 100 person-yrs | 95% CI | N | Events | Number of  progression per 100 person-yrs | 95% CI | N | Events | Number of  progression per 100 person-yrs | 95% CI |
| Overall |  | 594 | 57 | 0.87 | 0.67-1.12 | 301 | 34 | 1.01 | 0.73-1.42 | 293 | 23 | 0.72 | 0.48-1.08 |
| Age at screening | <65 years | 157 | 29 | 1.09 | 0.76-1.57 | 96 | 21 | 1.34 | 0.87-2.06 | 61 | 8 | 0.74 | 0.37-1.47 |
|  | ≥65 years | 437 | 28 | 0.71 | 0.50-1.04 | 205 | 13 | 0.73 | 0.42-1.26 | 232 | 15 | 0.70 | 0.43-1.17 |
| Isotype* | IgG | 412 | 30 | 0.62 | 0.54-0.88 | 206 | 19 | 0.75 | 0.48-1.17 | 206 | 11 | 0.45 | 0.25-0.81 |
|  | Non-IgG | 168 | 26 | 1.70 | 1.16-2.49 | 84 | 14 | 1.70 | 1.01-2.86 | 84 | 12 | 1.41 | 0.80-2.47 |
|  | IgM | 102 | 11 | 1.21 | 0.67-2.17 | 50 | 5 | 1.04 | 0.44-2.49 | 52 | 6 | 1.17 | 0.52-2.59 |
|  | IgA | 66 | 15 | 2.44 | 1.48-4.03 | 34 | 9 | 2.63 | 1.38-5.01 | 32 | 6 | 1.76 | 0.80-3.89 |
| M protein | Normal | 432 | 33 | 0.62 | 0.45-0.88 | 222 | 22 | 0.80 | 0.53-1.21 | 210 | 11 | 0.44 | 0.24-0.79 |
|  | Abnormal | 124 | 22 | 1.51 | 1.00-2.28 | 63 | 11 | 1.53 | 0.85-2.75 | 61 | 11 | 1.48 | 0.83-2.67 |
| Free light-chain ratio (FLCr) | Normal | 375 | 25 | 0.58 | 0.39-0.86 | 190 | 17 | 0.75 | 0.47-1.20 | 185 | 8 | 0.37 | 0.19-0.75 |
|  | Abnormal | 145 | 28 | 1.89 | 1.31-2.72 | 72 | 13 | 1.61 | 0.94-2.76 | 73 | 15 | 1.79 | 1.08-2.96 |
| M protein and FLCr | Both M protein  and FLCr normal | 295 | 17 | 0.47 | 0.29-0.76 | 148 | 12 | 0.64 | 0.37-1.13 | 147 | 5 | 0.29 | 0.12-0.69 |
|  | Elevated M  protein only | 64 | 7 | 0.96 | 0.46-2.00 | 33 | 4 | 1.06 | 0.40-2.82 | 31 | 3 | 0.84 | 0.27-2.59 |
|  | FLCr abnormal only | 88 | 15 | 1.36 | 0.82-2.24 | 47 | 9 | 1.53 | 0.80-2.93 | 41 | 6 | 1.15 | 0.52-2.56 |
|  | Both abnormal | 41 | 12 | 2.47 | 1.41-4.32 | 19 | 4 | 2.07 | 0.79-5.47 | 22 | 8 | 2.73 | 1.38-5.41 |
| Isotype, M protein, FLCr | IgG type, M protein and FLCr normal | 209 | 11 | 0.41 | 0.22-0.73 | 102 | 8 | 0.59 | 0.30-1.18 | 107 | 3 | 0.22 | 0.07-0.68 |
|  | Any 1 abnormal | 178 | 16 | 0.78 | 0.48-1.27 | 92 | 10 | 0.91 | 0.49-1.69 | 86 | 6 | 0.62 | 0.28-1.38 |
|  | Any 2 abnormal | 77 | 15 | 1.71 | 1.03-2.82 | 39 | 7 | 1.70 | 0.82-3.54 | 38 | 8 | 1.71 | 0.86-3.41 |
|  | Any 3 abnormal | 14 | 8 | 5.56 | 2.83-10.9 | 6 | 3 | 5.88 | 1.96-17.6 | 8 | 5 | 5.38 | 2.29-12.6 |
| Body Mass Index (kg/m^2^) | <25 | 181 | 9 | 0.47 | 0.25-0.90 | 73 | 6 | 0.74 | 0.33-1.64 | 108 | 3 | 0.27 | 0.09-0.85 |
|  | ≥25 | 374 | 47 | 1.03 | 0.77-1.36 | 209 | 27 | 1.07 | 0.74-1.56 | 165 | 20 | 0.97 | 0.63-1.50 |

*Biclonal gammopathy were excluded from all the analyses that were performed according to isotype

**Supplementary Table 2.** Sensitivity analysis for Cox regression models for risk of MGUS progression by BMI categories

|  |  | Univariate | | | Univariate** | | | Multivariable** | | |
| --- | --- | --- | --- | --- | --- | --- | --- | --- | --- | --- |
|  |  | HR | 95% CI | P | HR | 95% CI | P | HR* | 95% CI | P |
| BMI (≥25) | Overall | 2.14 | 1.05-4.36 | 0.04 | 2.07 | 0.97-4.42 | 0.06 | 1.92 | 0.90-4.11 | 0.09 |
|  | Male | 1.39 | 0.57-3.36 | 0.47 | 1.21 | 0.46-3.21 | 0.70 | 1.13 | 0.42-2.99 | 0.81 |
|  | Female | 3.55 | 1.06-11.9 | 0.041 | 3.49 | 1.03-11.8 | 0.044 | 3.46 | 1.02-11.8 | 0.047 |
|  |  |  |  |  |  |  |  |  |  |  |
| BMI per 5 units | Overall | 1.24 | 0.98-1.58 | 0.07 | 1.28 | 1.00-1.65 | 0.049 | 1.23 | 0.94-1.60 | 0.13 |
|  | Male | 1.17 | 0.82-1.68 | 0.39 | 1.25 | 0.84-1.84 | 0.27 | 1.12 | 0.75-1.68 | 0.57 |
|  | Female | 1.31 | 0.94-1.83 | 0.11 | 1.31 | 0.94-1.83 | 0.11 | 1.49 | 1.02-2.17 | 0.04 |

*Adjusted for age at MGUS screening date, Isotype, M protein, and FLCr.

**For overall: model includes 446 MGUS cases (with 49 events) who had all clinical data available; For male: model includes 223 MGUS cases (with 27 events) who had all clinical data available; For female: model includes 223 MGUS cases (with 22 events) who had all clinical data available

**Supplementary Table 3.** Cox regression models for risk of MGUS progression

|  |  |  |  | Univariate | | | Univariate* | | | | | Multivariable** | | |
| --- | --- | --- | --- | --- | --- | --- | --- | --- | --- | --- | --- | --- | --- | --- |
|  |  | N | Event | HR | 95% CI | P | N | Event | HR | 95% CI | P | HR* | 95% CI | P |
| BMI (≥25) | Overall | 555 | 56 | 2.14 | 1.05-4.36 | 0.04 | 446 | 49 | 2.07 | 0.97-4.42 | 0.06 | 2.37 | 0.99-5.67 | 0.053 |
|  | Male | 282 | 33 | 1.39 | 0.57-3.36 | 0.47 | 223 | 27 | 1.19 | 0.40-3.51 | 0.76 | 1.38 | 0.46-4.13 | 0.57 |
|  | Female | 273 | 23 | 3.55 | 1.06-11.9 | 0.041 | 223 | 22 | 4.27 | 0.99-18.5 | 0.044 | 4.57 | 1.04-20.2 | 0.045 |
|  |  |  |  |  |  |  |  |  |  |  |  |  |  |  |
| Isotype (non-IgG) | Overall | 580 | 56 | 2.84 | 1.68-4.80 | 0.0001 | 446 | 49 | 3.22 | 1.83-5.65 | <0.0001 | 2.76 | 1.43-5.32 | 0.002 |
|  | Male | 290 | 33 | 2.50 | 1.25-4.99 | 0.01 | 223 | 27 | 2.67 | 1.14-6.25 | 0.024 | 1.93 | 0.79-4.76 | 0.15 |
|  | Female | 290 | 23 | 3.44 | 1.52-7.82 | 0.003 | 223 | 22 | 6.45 | 2.53-16.5 | <0.0001 | 4.48 | 1.63-12.3 | 0.004 |
|  |  |  |  |  |  |  |  |  |  |  |  |  |  |  |
| M protein (≥1.5 g/dl) | Overall | 556 | 55 | 2.57 | 1.50-4.42 | 0.001 | 446 | 49 | 2.50 | 1.41-4.45 | 0.002 | 2.80 | 1.44-5.45 | 0.003 |
|  | Male | 285 | 33 | 2.03 | 0.99-4.20 | 0.055 | 223 | 27 | 1.60 | 0.62-4.09 | 0.33 | 1.81 | 0.65-5.02 | 0.26 |
|  | Female | 271 | 22 | 3.64 | 1.57-8.41 | 0.003 | 223 | 22 | 4.16 | 1.69-10.2 | 0.002 | 4.14 | 1.61-10.6 | 0.003 |
|  |  |  |  |  |  |  |  |  |  |  |  |  |  |  |
| FLC_r_ (Abnormal) | Overall | 520 | 53 | 3.39 | 1.98-5.82 | <0.0001 | 446 | 49 | 3.49 | 1.99-6.12 | <0.0001 | 2.15 | 1.11-4.17 | 0.024 |
|  | Male | 262 | 30 | 2.32 | 1.13-4.77 | 0.023 | 223 | 27 | 2.60 | 1.13-6.01 | 0.025 | 1.77 | 0.73-4.29 | 0.21 |
|  | Female | 258 | 23 | 5.72 | 2.42-13.5 | <0.0001 | 223 | 22 | 6.25 | 2.37-16.5 | <0.0001 | 3.70 | 1.29-10.6 | 0.015 |
|  |  |  |  |  |  |  |  |  |  |  |  |  |  |  |
| Reduction (any) | Overall | 410 | 45 | 4.90 | 2.71-8.86 | <0.0001 | 358 | 41 | 5.41 | 2.92-10.0 | <0.0001 | 3.77 | 1.92-7.41 | <0.0001 |
|  | Male | 206 | 25 | 5.29 | 2.40-11.7 | <0.0001 | 177 | 22 | 5.73 | 2.47-13.3 | <0.0001 | 5.00 | 2.06-12.1 | <0.0001 |
|  | Female | 199 | 20 | 4.41 | 1.79-10.9 | 0.001 | 168 | 19 | 4.97 | 1.99-12.4 | 0.001 | 1.83 | 0.63-5.34 | 0.27 |
|  |  |  |  |  |  |  |  |  |  |  |  |  |  |  |

*Also adjusted for age at MGUS screening date.

**For overall: model includes 358 MGUS cases (with 41 events) who had complete clinical data available; For male: model includes 177 MGUS cases (with 22 events) who had complete clinical data available; For female: model includes 168 MGUS cases (with 19 events) who had complete clinical data available

**Supplementary Table 4.** Cox regression models for risk of MGUS progression by BMI at baseline and follow-up (or diagnosis)

|  | Adjusted for age and sex | | | | Adjusted for age, sex, BMI at baseline | | Adjusted for age, sex, isotype, FLCr, M protein | | Adjusted for age, sex, isotype, FLCr, M protein, BMI at baseline | |
| --- | --- | --- | --- | --- | --- | --- | --- | --- | --- | --- |
| **BMI categorical Change** Overall | N | Event | HR | 95% CI | HR* | 95% CI | HR** | 95% CI | HR** | 95% CI |
| BMI stays <25 both time points | 123 | 7 | 1 | ref | 1 | ref | 1 | ref | 1 | ref |
| BMI >25 at baseline and BMI < 25 at follow-up | 101 | 5 | 0.66 | 0.21-2.07 | 0.69 | 0.20-2.33 | 0.51 | 0.15-1.76 | 0.49 | 0.14-1.80 |
| BMI <25 at baseline BMI >25 at follow-up | 21 | 2 | 1.17 | 0.24-5.70 | 1.18 | 0.24-5.77 | 0.57 | 0.07-4.79 | 0.57 | 0.07-4.78 |
| BMI High at both time points. | 193 | 40 | 2.73 | 1.20-6.24 | 2.92 | 1.08-7.91 | 2.40 | 1.03-5.56 | 2.27 | 0.83-6.21 |

*N=434; **N=362
